# Supplementary material for: Changes and predictors of premarital sex intercourse among never-married women (15–24 years) in Nigeria: A multilevel approach
Source: PLoS One. 2025 Apr 29;20(4):e0321783. doi: 10.1371/journal.pone.0321783 (PMC12040172; doi:10.1371/journal.pone.0321783)
Supplement: Attachment — Submitted filename: Response to Reviewers.docx [file pone.0321783.s002.docx]

Dear Nega Degefa Megersa, Msc
Academic Editor
PLOS ONE

**Re:**PLOS ONE Decision: Revision required [PONE-D-24-33075] - [EMID:4f2e04d4c21a5c45]

**Changes and predictors of premarital sex intercourse among never-married women (15-24 years) in Nigeria: A multilevel approach**

**A rebuttal letter**

We thank the reviewers and editors for their time and comments on this manuscript. We have edited the manuscript to address their concerns. We believe the manuscript has addressed the points raised during the review process and is now suitable for publication in PLOS ONE. Response to each point raised by the academic editor and reviewers are as follows:

**Journal Requirements:**

When submitting your revision, we need you to address these additional requirements.

**Comment 1:** Please ensure that your manuscript meets PLOS ONE's style requirements, including those for file naming. The PLOS ONE style templates can be found at 
<https://journals.plos.org/plosone/s/file?id=wjVg/PLOSOne_formatting_sample_main_body.pdf>  and 
<https://journals.plos.org/plosone/s/file?id=ba62/PLOSOne_formatting_sample_title_authors_affiliations.pdf>

**Response: Thank you. The PLOS ONE’s style requirements (i.e. Title, Author, Affiliations Formatting Guidelines and Manuscript Body Formatting Guidelines) have been strictly followed.**

**Comment 2:** PLOS requires an ORCID iD for the corresponding author in Editorial Manager on papers submitted after December 6th, 2016. Please ensure that you have an ORCID iD and that it is validated in Editorial Manager. To do this, go to ‘Update my Information’ (in the upper left-hand corner of the main menu), and click on the Fetch/Validate link next to the ORCID field. This will take you to the ORCID site and allow you to create a new iD or authenticate a pre-existing iD in Editorial Manager.

**Response: Thank you, ORCID ID (http://orcid.org/0000-0002-5645-7689)** **has been validated in Editorial Manager.**

**Comment 3:** Your ethics statement should only appear in the Methods section of your manuscript. If your ethics statement is written in any section besides the Methods, please delete it from any other section.

**Response: Thank you. As suggested, the Ethics statement that appears in any section besides the methods Section has been deleted. Ethics statements now appear only in the Methods section of the Manuscript.**

**Additional Editor Comments:**

**Abstract**

**Comment 1:** The abstract is well-written and concisely summarized. Limit the abbreviations in the abstract section. Thus, put IR, ICC, and PCV in full on their initial appearance**.**

**Response: Thank you. As suggested, the abbreviations in the abstract** **have been corrected. IR has been replaced with Individual recode, ICC has been replaced with Intra-Class Correlation, and PCV has been replaced with Proportional Change in Variance.**

**Comment 2:** The prevalence of PSI over ten years was described as 31.8%, consider 95% CI to provide a more complete picture of the problem by quantifying uncertainty, aiding in comparisons, informing decision-making, and evaluating the reliability of estimates.

**Response: Thank you. Agreed. As suggested 95%CI has been included.**

**Introduction**

**Comment:** Thus, this study could inform the development of policy on premarital sexual intercourse among young women in Nigeria. How this study could inform policy and what areas should the policy address to further hasten the declining PSI rate in Nigeria?

**Response: Thank you for your suggestion. An area to further hasten the declining PSI rate in Nigeria has been added.**

**Method**

**Comment:** Missing values on age at first sex are considered as not having had sexual intercourse and are included in both the numerator and denominator. What does this mean, would you please explain it? What techniques have you employed to manage your missing values?

**Response: Thank you. In the DHS, a missing value means that the variable is not applicable or there was not a valid response. Thus, we handled the missing data by declaring them as missing. Thus, dropping/removing the values/data/observation from the analysis.**

**Conclusion**

**Comment 1:** The conclusion appears to be well-supported by the study's data. However, a more comprehensive analysis, addressing the points mentioned below, would enhance the conclusion. While a decrease in prevalence is observed, it's crucial to assess the statistical significance of this trend. Is the decline statistically significant, or could it be due to chance?

**Response: Thank you for your suggestion. The following statement has been added to read: ‘While a decrease in prevalence is observed, it's crucial to assess the statistical significance of this trend. Is the decline statistically significant, or could it be due to chance?)’**

**Comment 2:** Acknowledging the limitations of the research, strengthens the credibility of the conclusion. It is beneficial if specific predictors of premarital sexual intercourse are included instead of referring to predictors of PSI in general. It could be strengthened by explicitly outlining specific policy recommendations based on the study's findings, which would make the research more doable.

**Response: Thank you for providing this insight. Predictors of premarital sexual intercourse have been included.**

**Comments to the Author**

**Reviewer #1: Obasanjo Bolarinwa**

Research Gap:

1. **Comment:** The introduction outlines the consequences of premarital sexual intercourse (PSI) among young women, but it does not sufficiently highlight the novel contribution of this study. While the authors mentioned that existing studies focus on adolescents, there is a need to emphasize the lack of longitudinal data or multilevel approaches in previous research. The text should explicitly state how this study fills the gap by using 10-year pooled data and a multilevel binary logistic regression model to track PSI trends over time.

**Response: Thank you. It has been added to read:**

‘Most of these studies on PSI have documented a range of individual-level factors such as; respondent’s age, region of residence, place of residence, wealth status, employment status, religion, and education among others as predictors of PSI. To the best of our knowledge, there is a paucity of information on studies on community-level factors on PSI in Nigeria. In addition to this, there is a dearth of studies on the general change in premarital sexual intercourse among young women using longitudinal nationally representative data, to examine the progress made in reducing PSI in Nigeria. Thus, this study will fill this knowledge gap by using pooled nationally representative data and a multilevel binary logistic regression model to track PSI trends and their predictors over time in Nigeria. The use of 15-year duration of 3- waves NDHS data offers new insights that previous studies could not. Thus, this study aimed to investigate changes and predictors of premarital sexual intercourse among young women. Assessing the changes and predictors of PSI could inform the development of policies and programmes on PSI among young women in Nigeria that will empower young women to make informed choices about their sexual and reproductive health rights.’

**Suggested Improvements:**

- **Comment:** • Line 56-61:

Strengthen the argument for why a multilevel approach is necessary for understanding PSI and why existing single-level analyses are insufficient. Discuss why previous studies lacked this dimension and how the author's study adds value.

- **Response: Thank you. It has been added as suggested (**See response to Research Gap comment 1 above**)**

**Specific Lines for Improvement:**

- **Comment:** Line 28-30:

Clarify the unique contribution of the author's work in comparison to previous studies, such as by stating, “This study is the first to use a multilevel analysis approach across a 10-year dataset to investigate changes in PSI and its predictors.”

- **Response: Thank you. It has been added (**See response to Research Gap comment 1 above**)**
- **Comment:** Line 72-75: Expand on the limitations of prior research by clearly stating the gaps in the context of multilevel modeling and the lack of longitudinal studies, especially in Nigeria.
- **Response: Thank you. Limitations of prior research in multilevel and longitudinal studies have been expanded as suggested (**See response to Research Gap comment 1**)**
- **Comment:** Line 102: Further justification is needed for using this particular dataset and why the pooled 10-year NDHS data offers new insights that previous studies could not.
- **Response: Thank you. Justification for using a 15-year duration of 3 waves NDHS has been added (**See response to Research Gap above**).**

**Grammatical Issues and Improvements:**

- **Comment:** Line 25: "a multilevel approach" – should be rephrased to "using a multilevel approach" for clarity.
- **Response: Thank you for your suggestion. It has been corrected to read** "using a multilevel approach"
- **Comment:** Line 78: "Prevalence of PSI decreased steadily" – consider rephrasing to "The prevalence of PSI steadily decreased" to improve readability.
- **Response: Thank you for your suggestion. It has been corrected to read** "The prevalence of PSI steadily decreased"
- **Comment:** Line 120-122: "The ICC value of 10.1% and the PCV of 41.3% show that the individual and community factors were important in explaining the variations in PSI." – This sentence can be made more concise and clear. Suggested: "An ICC value of 10.1% and a PCV of 41.3% indicate that both individual and community factors significantly influenced PSI variations."

**Response: Thank you for your suggestion. It has been corrected as suggested.**

- **Comment:** Line 180: "empower young women to achieve a further drop in PSI" – should be revised to "empower young women to contribute to a further reduction in PSI" for a more academic tone.
- **Response: Thank you for your suggestion. It has been corrected to read: ‘**to empower young women to contribute to a further reduction in PSI to improve the general national health status of women and to ensure progress towards achieving a reduction in early pregnancies among unmarried women’

**Methodological Issues and Suggestions for Improvement:**

- **Comment:** Line 32-35: The rationale for pooling data from the 2008, 2013, and 2018 surveys is briefly mentioned. However, it is crucial to provide more justification for why pooling these datasets is appropriate for studying changes in PSI over time. A clearer explanation of how trends in these periods will inform the research objectives would strengthen the methodology.
- **Response: Thank you. That is an interesting query. It has been added to read. ‘**The datasets were pooled to increase the sample size and enable studying changes in PSI over time’.
- **Comment:** Line 67-70: The study design description should expand on how cross-sectional data limits the ability to infer causality. Cross-sectional studies are limited in their ability to determine temporal relationships, which should be explicitly acknowledged in the limitations.
- **Response: Thank you. The Limitation of a cross-sectional study design has been acknowledged and incorporated.**
- **Comment:** Line 80-85: The method used for handling missing data is not well elaborated. It would benefit the manuscript to detail if any imputation methods were applied to handle missing values or whether complete case analysis was used.
- **Response: Thank you. Missing data were dropped. A complete case was deleted in the data analysis.**
- **Comment:** Line 90-92: The explanation of multilevel modeling is good, but the manuscript would benefit from including a brief description of why this modeling technique was chosen, particularly regarding the hierarchical structure of the data (i.e., individual and community levels).
- **Response: Thank you. It has been added to read:** ‘Finally, owing to the hierarchical nature of the NDHS data and the objective of this study, a four-model multi-level binary logistic regression was constructed to examine the individual and community level factors associated with PSI’.
- **Comment:** Line 102: It is important to elaborate on the rationale behind selecting the specific contextual variables for inclusion in the model. While it is mentioned that they are based on previous literature, more detail is needed on how these factors were prioritized and why others may have been excluded.
- **Response: Thank you. It has been added to read:**

Odds ratio (OR) and 95% confidence intervals (CI) were provided for all models in the multivariable analysis while multicollinearity was done using variance inflation factor (VIF) to exclude explanatory variables with evidence of collinearity.

- **Comment:** Line 112-115: The results of the multicollinearity test (variance inflation factor, VIF) are mentioned but not fully explained. The manuscript should include what threshold of VIF was used to determine multicollinearity and how any problematic variables were addressed.
- **Response: Thank you for providing these insights**. **It has been added to read:** ‘A Multicollinearity test was performed using variance inflation factor (VIF < 5). All explanatory variables with evidence of no collinearity (mean VIF= 1.38, maximum = 1.87 and minimum VIF = 1.10) were retained in the models while those with evidence of collinearity (VIF > 5) were excluded from the models.’

*********************************************************************************

**Reviewer #2: Phillips Edomwonyi Obasohan**

Changes and predictors of premarital sex intercourse among never-married women (15-24 years) in Nigeria: A multilevel approach
The author has addressed an important topic of social and public health interest. The prevalence of PSI globally has been identified to be high and could result in unwanted pregnancy, school dropout among females, sexually transmitted diseases, etc. But I have the following observations and comments, which the author may wish to consider:

- **Abstract**
  **Comment:** You have stated that the ICC of 10.1% and PCV of 48.3% imply that individual and community factors are important. Do you mean the ICC explained the importance of individual factors, while the PCV explains the importance of community factors? This may be confusing to some readers.
- **Response: Thank you. That is an interesting query. We have incorporated your comments to read:** The Intraclass correlation coefficient (ICC) explained the proportion of total variance in the PSI that is attributable to the area level while the proportional change in variance (PCV) explained the variability in the odds of PSI explained by each of the models Therefore, ICC of 10.0% shows that the individual and community factors were important in explaining the variations in PSI between clusters. The PCV of 41.3% explained the variability in the odds of PSI explained by each of the models with more terms.

**Background study**
**Comment 1:** The topic covers the changes and the predictors of PSI, but you have discussed what literature found on changes and prevalence without reference to what previous literature found on the predictors.

**Response: Thank you for this observation. As suggested, it has been added to read: ‘**Most of these studies on PSI have documented a range of individual-level factors such as; respondent’s age, region of residence, place of residence, wealth status, employment status, religion, and education among others as predictors of PSI’.

**Comment 2:** There were no explicit stated aims and objectives in the study.

**Response: Thank you. We have elaborated on it** **to read: ‘**Thus, this study aimed at investigating changes and predictors of premarital sexual intercourse among young women’

**Methods**
Data source
**Comment 1:** You have stated you used a 10-year pooled data set. Do you have any specific reasons for choosing 10 years out of the existing six waves that have spanned over 30 years?

**Response: Thank you for noting this.** The study used the last three waves of NDHS to map out major trends in the country focusing on young women and to evaluate the extent of various national variations in PSI. Pooling data provides an opportunity to increase the number of observations, strength of evidence, and analyse of a much larger data set. It makes it possible to access diverse data and perform analysis on data that is updated regularly. It enhanced statistical power, the ability to compare outcomes and validate models across sites or settings, and opportunities to develop new measures. The large size of the data enhances the quality of the original studies that were combined, the statistical methods used for the pooled analysis. It increased study power that permits a full examination of effect modification within the data.

**Comment 1:** The three data sets actually spanned over 15 years of the duration, with 3 waves. I am not sure they are '10-year waves' as stated. For instance, the 2008 wave covered 2003-2008, 2013 covered 2008-2013, and 2018 covered 2013-2018. This is a 15-year duration of 3 waves (2008, 2013, and 2018).

**Response: Thank you for providing this insight.** It **is an interesting query. I agree with you and have incorporated this suggestion throughout the manuscript to read: “A 15-year duration of 3 waves (2008, 2013, and 2018) NDHS datasets”.**

**Outcome variables**
**Comment 1:** What does ‘never unmarried’ mean? This may be a typo-error. Check it

**Response: Yes. Thank you for the observation. It has been corrected to read “never married”**

**Comment 2:** How did you handle the missing data?

**Response: Thank you. In the DHS, missing means the variable is not applicable or there was not a valid response. Thus, we handled the missing data by dropping/removing the values/data/observations from the analysis or declaring them as missing.**

**Predictors**
**Comment:** I suppose that the unit of analysis from this study is ‘never-married’ women aged 15 to 24 years. The outcome variable, PSI, is an onset at a point in time. I am wondering how some individual characteristics that came to be after the onset of the outcome can be considered a predictor or a determinant of the status (PSI). For instance, a partner's education status, parity, child mortality, etc. Moreover, some of these predictors were listed but were missing in the analysis.

**Response: Thank you. Yes, they were dropped eventually from the analysis. Thus,** It has been removed the text.

**Statistical Analyses**
**Comment 1:** I am wondering if STATA 14 could handle some features in mixed effect analysis. Many updates have occurred since Stata 14 was released. Can you trust the results you got? In addition, I am not sure if Stata 14 could handle some features in NDHS 2018. Please verify this.

**Response: Yes. Stata 14 handled it perfectly.**

**Comment 2:** Rewrite the ICC and PCV formulas in equation format if you need to keep them in the paper.

**Response: Thank you. They have been corrected as:**

$ICC= \frac{VA}{(VA + 3.29)} x 100$ and	$PCV= \frac{VA - VB}{VA} x 100$

**Handling missing values statement**

**Comment 1:** What do you mean by ‘numerator’ and 'denominator’?

**Response: Thank you. It is confusing. It has been deleted.**

2. **Comment:** Was age the only variable with missing values?

**Response: Thank you. No. Age at first sex was not the only variable with missing values.**

3. **Comment:** How were the missing values in other variables handled? In other words, what was the magnitude of missingness in the data?

**Response: Thank you. Missing values in other variables (marital status = 1 (0.0%), religion= 114 (0.48%), working status= 158 (0.66%), mass media: radio=13 (0.05%), TV = 18 (0.08%), newspaper= 39 (0.16%), community literacy= 68 (0.29%)** **were declared as missing values and removed from the analysis. In this manuscript, the magnitude of missingness in the data is less than 1%.**

**Results**
**Comment 1:** Table 2: Why was the chi-sq for age in 2008 having a triple star (***)?

**Response: Thank you. It has been deleted.**

**Fixed effects**
**Comment 2:** The reporting style here is confusing. You applied multilevel analysis (having fixed effect and random effect components). Under the ‘fixed effect’ heading, you reported the random effect, while almost nothing was reported under ‘random effect.’

**Response: Thank you for this important observation. It has been reorganised**

**Discussion**
**Comment 1:** This statement lowers the odds of PSI among Islamic women over other religions. You said the reason is that ‘the teaching and practices of Islam frown upon PSI’. This is controversial because other religions (i.e., Christianity) also teach and frown at PSI. Do you have references to support your claims?

**Response: Thank you. It has been corrected to read: ‘Previous studies have reported lower odds of premarital sex among Muslim girls compared to Christians and Hindus. It has been asserted that religion has a powerful influence on sexual behaviour with Islam being the most successful religion in putting forth religious precepts about premarital sex and getting its adherents to abide by these precepts. Other factors that might have influenced the finding include age at marriage for Muslim girls, restriction on women’s mobility and the fact that Muslims are less likely to report honestly about their transgression if they had engaged in PSI than other religious affiliations.**’

**Comment 2:** Some of the predictors of PSI listed in the paper were not used in the analysis and were not discussed. For instance, ‘partner’s education' and ‘ever experience child mortality’ were listed. However, 'sex of household head’ could be a confounding variable but was not included in the analysis.

**Response: Thank you for the observation. As shown in all the Tables, the mentioned predictors were dropped from the study. Thus, the variables (**partner’s education and ‘ever experience child mortality’ etc) **have been removed from the explanatory variables section and variables used in the manuscript. In addition, the omitted variable (community literacy) has been included. Also, the sex of the household head’ could be a confounding variable but was not included in the analysis. It could be investigated in other future studies.**

**Conclusion**
**Comment 1:** Too brief

**Response:** We have redrafted the section to establish a clearer focus

**Comment 2:** What were the implications of the findings, and could they be used to improve the PSI situation in Nigeria?

**Response:** We have redrafted the section to established a clearer focus

**Comment 3:** Make a statement on the direction of future study.

**Response: Thank you. It has been corrected to read:**

‘The study concludes that even though a steady decrease in the prevalence of PSI among never-married women (15-24) over the fifteen-year duration of three waves of NDHS datasets (2008, 2013 and –2018) had been observed, it’s crucial to assess the statistical significance of this trend in further study. Is the decline statistically significant, or could it be due to chance? Furthermore, the study has identified some predictors of PSI among never-married women (15-24). Factors such as age, education, religion, wealth status, media exposure, region of residence, community literacy level, knowledge of contraceptives, and socio-economic status were identified to be associated with PSI. This study has policy implications. There is a need for policies and programmes that will factor in these predictors in promoting young women’s reproductive and health rights in Nigeria. Therefore, the study calls for concerted efforts that will be well tailored to achiev**e** further drops in PSI to thereby improve the general national health status of women.’

Finally, I thank you for allowing me to strengthen this manuscript with your valuable comments and queries. I have worked hard to incorporate your feedback and hope that these revisions persuade you to accept our submission.

Yours sincerely,

Dr. Joseph A. Kupoluyi

Demography and Social Statistics.
