# Supplementary material for: Changes and predictors of premarital sex intercourse among never-married women (15–24 years) in Nigeria: A multilevel approach
Source: PLoS One. 2025 Apr 29;20(4):e0321783. doi: 10.1371/journal.pone.0321783 (PMC12040172; doi:10.1371/journal.pone.0321783)
Supplement: Attachment — Submitted filename: RESPONSE_TO_REVIEWERS_auresp_2.docx [file pone.0321783.s003.docx]

**Major Concerns:**

1. **Methodological Issues:**

**Query 1: The handling of missing data requires more detailed explanation. While the authors mention excluding missing values, a more thorough discussion of the potential impact of these exclusions on the results is needed.**

Answer: Thank you for this observation. The percentage of missing data is low (less than 1). Therefore, missing data has no practical effect on the analysis. Also, to ensure that statistical estimates based on DHS data are valid, as recommended by DHS, sample weights and STATA survey command (svy) command in the analysis were applied to adjust for the over/under-sampling and non-response during the survey.

**Query 2:** **The potential for social desirability bias in self-reported sexual behaviour data should be more thoroughly addressed in the limitations section.**

Answer: Thank you for this observation. The potential for social desirability bias in self-reported sexual behaviour data has been addressed in the limitations section. It now read: ‘In addition to the weakness of the dataset is the fact that DHS information on sexual behaviour was collected through a self-reporting of retrospective events. Thus, there is the possibility of over-reporting or under-reporting of cases of PSI in this study. Therefore, the study should be interpreted with caution based on the perceived social norms and culture.

2. **Analytical Concerns:**

**Query 1: The statistical significance of the observed temporal trends in PSI requires more rigorous testing. While the author note a declining trend, formal tests for trend should be included.**

Answer: Thank you for this observation. Trend detention in time was conducted using Mann Kendall test. It is a test that helps us know whether a trend exists in a time and whether it is upward or downward and statistically significant. In this study, the trend is monotonic downward and significant (p < 0.001). This has been specified in the manuscript.

**Query 2: The interaction effects between individual and community-level factors warrant exploration.**

Answer: Thank you. Interaction effect analyses between individual and community-level factors implies another level of analysis which future study could explore. However, this has been explained further.

3. **Results Presentation:**

**Query 1: The presentation of results could be improved through clearer organization of the findings section, more consistent reporting of confidence intervals, and more detailed interpretation of the random effects results.**

Answer: Thank you. The result section has been improved. All confidence intervals has been reported. Also, more detailed interpretation of the random effects results has been done.

4. **Specific Recommendations:**

**Introduction:**
**Ouery 1: The rationale for the multilevel approach needs strengthening, along with more context about Nigeria's sexual and reproductive health policies.**

Answer: Thank you for this observation. The rationale for multi-level has been strengthen to read:

‘Community-level factors refer to different characteristics of the social group to which individual belong. These characteristics may affect PSI either directly or indirectly by controlling how individuals’ characteristics affect PSI. It is therefore important to know the effect of community-level variables on individual–level and household-level outcomes on PSI. The availability of DHS hierarchical data in which individuals are clustered within household and household clustered within the communities, give rooms to account for the effect of both the individual and group-level influence on PSI in Nigeria’.

Furthermore, more context about Nigeria’s sexual and reproductive health policies have been included. It reads:

‘The national sexual and reproductive health policies has been anchored within the framework of the national health policy. The policy recognizes the implementation of reproductive health within the context .of primary health care, with the goal of ensuring availability and access to complete sexual and reproductive health information and quality of services. The policy largely discouraged PSI with a strongly but not openly promoting abstinence until marriage as ideal behaviour. Under the policy, PSI among young women aged 15-24 is considered illegal. However, the policy provides a comprehensive sex education and contraceptive services to young people to help them make informed decisions about sexual health, including preventing unwanted pregnancies and sexually transmitted infections (STIs). In addition, the policy accentuates the need for access to reproductive health services for those who are sexually active but do not aggressively criminalise premarital sex. The social and cultural norms around the policy vary depending on region and religious beliefs. Thus, making some of these policies and programmes largely ineffective’.

**Query 2: The theoretical framework should be better integrated with existing literature.**

Answer: Thank you. The theoretical framework has been included to read:

‘This study theoretical framework was drawn from the Theory of Planned Behaviour and Social Cognitive Theory. The theories emphasized on the role of individual attitudes in considering whether PSI is morally acceptable or not; feels of engaging in PSI or not (social norms), has access to contraception and personal confidence (perceived control), and has personal beliefs to influencing decisions about PSI, while considering factors such as family background, peer pressure, religious values, and personal commitment to a partner’.

5. **Methods:**

**Query 1: The author should provide more detail about the sampling strategy and power calculations.**

Answer: Thank you. More details about the sampling strategy and power calculations has been added. It now read: ‘DHS used a two-stage sampling procedure to collect data from all the 36 states and the federal capital territory (FCT), Abuja, Nigeria. The samples for the survey were drawn randomly from cluster or enumeration areas (EAs). The EAs served as the primary sampling unit for the survey. A systematic selection of households from the list of households were done to interview eligible respondents. Women aged 15-49 and men aged 15-64 were selected systematically from the households and interviewed’.

**Query 2: The construction of community-level variables needs clarification.**

Answer: Thank you. The construction of all the community level variables has been added to read:

‘--- a combined frequencies of reading newspaper, watching television and listening to radio within a week. The scores were further distributed into two groups (exposed and not exposed). DHS measured wealth index as a uniform composite variable determined through principal component analysis (PCA) and was built on household properties such as: water supply, television, electricity, radio, refrigerator, type of flooring, and type of vehicle. The scores were categorised into five quintiles, and each quintile represents a relative measure of a household’s socioeconomic status. Contextual variables considered in this study were community socioeconomic status, community knowledge of modern contraceptives, community literacy and region of residence. The region of residence was measured and divided into five geopolitical groups namely: South West, South East, North West, North East, North Central, and South-South. Other contextual variables were constructed and classified by aggregating individual-level factors at the cluster level using the median value as a cut-off point. These variables were: community socioeconomic status (low, moderate, high), community literacy (low, moderate, high), and community knowledge of modern contraceptives (low, moderate, high). The community socioeconomic status was generated from the combination of individual wealth quintile while community literacy was generated from the combination of individual literacy level. Community knowledge of modern contraceptives was generated from the combination of individual knowledge of any contraceptive methods’.

**Query 3: Sensitivity analyses for key assumptions should be included.**

Answer: Thank you for this observation. A Mann Kendall test was performed. The distribution with other variable show a fair distribution.

**Query 4: The choice of variables included in each model requires better justification.**

Answer: Thank you. It has been stated earlier in the manuscript (see explanatory variables section, line 1-4) that ‘All the explanatory variables used for this study were selected based on the established association reported in the previous studies with PSI and their level of significance (p < 0.05)’. A Multicollinearity test was performed using a variance inflation factor (VIF < 5). All explanatory variables with evidence of no collinearity (mean VIF= 1.38, maximum = 1.87 and minimum VIF = 1.10) were retained in the models while those with evidence of collinearity (VIF > 5) were excluded from the models.

6. **Results:**

**Query 1: Formal tests for temporal trends should be added to support the observed patterns.**

Answer: Thank you for this observation. Trend detention in time has been conducted using Mann Kendall test. The result shows that the data series has a statistically significant trend at p< 0.001

**Query 2: Interaction analyses between key variables would strengthen the findings.**

Answer: Thank you. Interaction analyses between key variables implies another level of analysis which further study could explore.

**Query 3: The random effects require more detailed interpretation.**

Answer: Thank you for this observation. More explanations of the random effects has been done.

**Ouery 4: The addition of predicted probabilities for key findings would enhance understanding of the results**.

Answer: Thank you for this observation. Predicted probabilities for the key findings have been added.

7. **Discussion:**

**Query 1: The comparison with other studies from similar contexts needs strengthening.**

Answer: Thank you for the observations. The comparison with other studies has been done to strengthen the manuscript.

**Query 2: Policy implications of the findings should be expanded.**

Answer: Thank you. Policy implications of the findings has been expanded.

**Query 3: The sustainability of observed improvements requires more thorough examination.**

Answer: Thank you. The sustainability of observed improvements has been examined and expanded.

**Query 4: The implications of the community-level effects warrant deeper discussion.**

Answer: Thank you. The implications of the community-level effects has been discussed.
